# Enzymatic Hydrolysis With Pepsin Enhanced the Nutrient Compositions of Unfractionated Soy Protein Hydrolysate and Its Cell Viability and Nitric Oxide Activities

**DOI:** 10.1002/fsn3.71147

**Published:** 2025-11-14

**Authors:** Oluwafemi Ayodeji Idowu, Chutha Takahashi Yupanqui

**Affiliations:** ^1^ Functional Food and Nutrition Program, Center of Excellence in Functional Foods and Gastronomy, Faculty of Agro‐Industry Prince of Songkla University Hat Yai Songkhla Thailand; ^2^ Biochemical and Toxicology Unit, Department of Environmental Health Science, Faculty of Health Sciences National Open University of Nigeria Abuja Nigeria

**Keywords:** amino acid composition, anti‐inflammation, antioxidant, enzyme hydrolysis, pepsin, soy protein hydrolysate

## Abstract

The crude soy protein isolate (SPI), a by‐product of soybean processing, is a valuable source of plant proteins but is limited by poor digestibility, low solubility, and restricted functionality due to its high molecular weight. Enzymatic hydrolysis offers an effective way to overcome these limitations. This study aimed to develop soy protein hydrolysate (SPH) using pepsin and evaluate its nutrient composition, antioxidant, and anti‐inflammatory activities. SPI was pre‐incubated and hydrolyzed with pepsin at an enzyme‐to‐substrate ratio of 1.5% (w/w) for 4 h. The molecular weights of SPI and SPH were determined by SDS‐PAGE. The nutrient composition, antioxidant (DPPH, FRAP, ORAC), cell viability, and the inhibitory effect of the SPH on nitric oxide‐mediated inflammation were evaluated using RAW‐264.7 cells. SDS‐PAGE analysis revealed that SPH had lower molecular weight peptides (< 20 kDa) compared to crude SPI (> 65 kDa). The hydrolysate showed a high protein recovery yield (89.70%) and consisted of 71.18% protein, 13.66% carbohydrates, 13.14% dietary fiber, 10.79% ash, 2.45% moisture, 1.92% fat, sodium (5496.63 mg), calcium (85.53 mg), and iron (6.54 mg), providing 356.64 kcal/100 g energy. Amino acid profiling indicated 45.36% essential and 54.70% non‐essential amino acids, with glutamate being predominant (18.35%). SPH exhibited significant (*p* < 0.05) radical scavenging activity and inhibited nitric oxide production in LPS‐stimulated RAW 264.7 cells at 10 mg/mL. It also enhanced cell viability by over 80% at 1, 2.5, and 10 mg/mL. These results suggest that pepsin‐derived SPH has enhanced nutritional quality and biological activity and could serve as a functional food ingredient to promote human health.

## Graphical abstract

This study examined protein hydrolysate derived from crude soy protein isolate through pepsin hydrolysis for improved nutrient composition, antioxidant, and anti‐inflammatory potential. Results showed that pepsin hydrolysis enhanced the nutrient and amino acid profiles of soy protein hydrolysate. The treatment also improved its ability to inhibit nitric oxide‐mediated oxidative stress and support cell viability. These findings highlight the potential of soy protein hydrolysate as a functional food ingredient for promoting animal and human health.

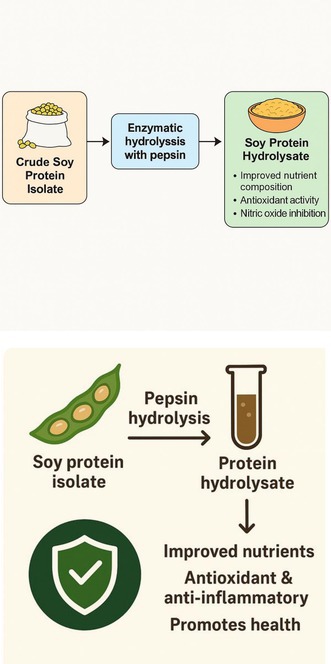

**Abbreviations**
!THAA: total hydrophilic amino acids
μg: microgram
Abs: absorbance
ANOVA: analysis of variance
DPPH: 1,1‐diphenyl‐2‐picrylhydrazyl
E/S: enzyme‐substrate ratio
EAA: essential amino acids
FRAP: ferric reducing antioxidant power
g: gram
Kcal: kilocalories
KDa: kilo Dalton
mg: milligram
ORAC: oxygen radical absorbance capacity
SDS‐Page: sodium dodecyl sulfate–polyacrylamide gel electrophoresis
SPHs: soil protein hydrolysates
SPI: soil protein isolate
TEAA: total essential amino acids
THAA: total hydrophobic amino acids
TNEAA: total non‐essential amino acids

## 1 Introduction

**FIGURE 1:**
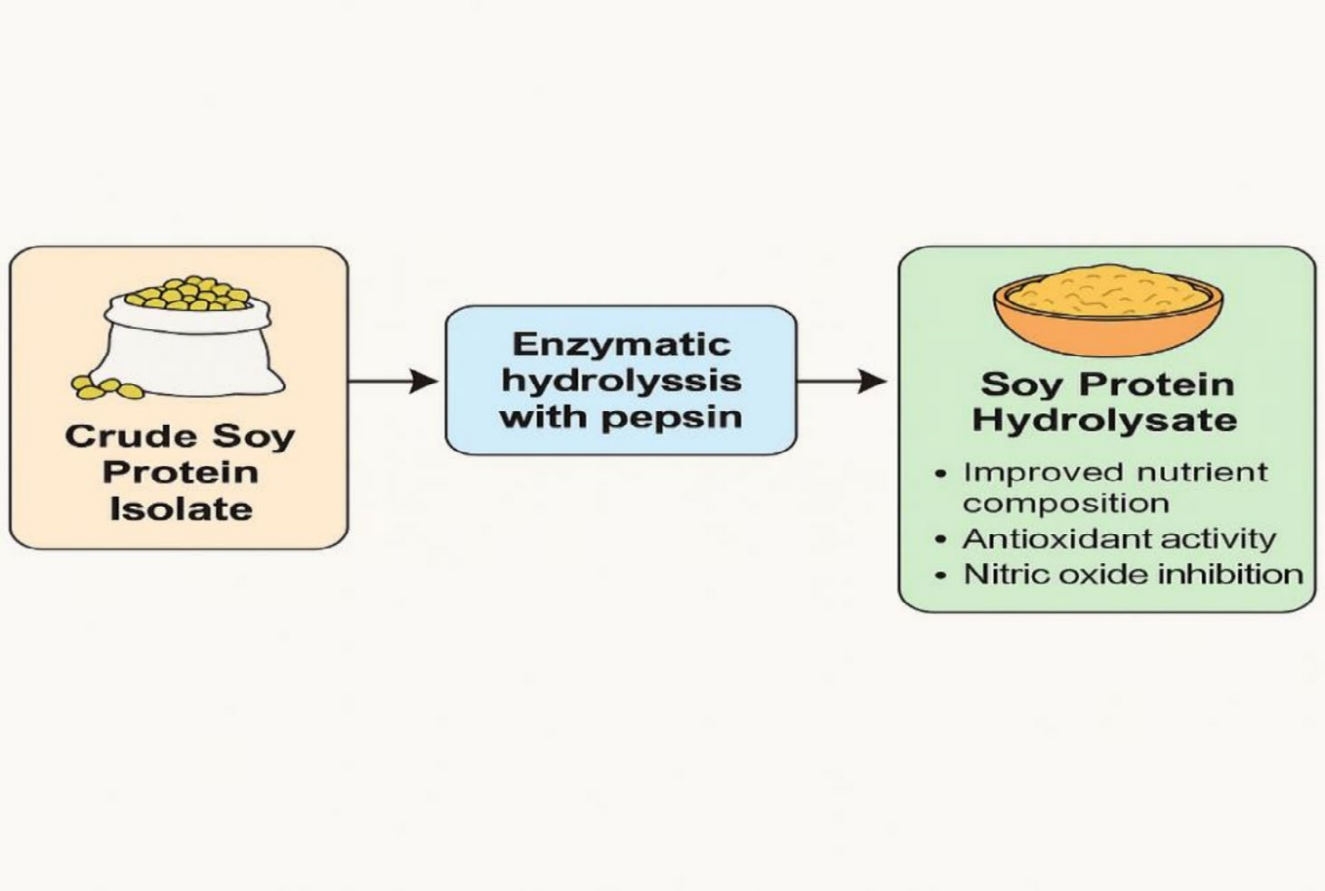
It represents Graphical abstract.

The need for food products made with fewer chemical methods is in high demand among consumers. Therefore, the application of protein hydrolysates in animal and human nutrition, food, and pharmaceutical industries as emerging functional foods is gaining much attention because of their excellent functional properties and health benefits. Soy protein is a protein obtained from soybeans ( *Glycine max* ). It contains 60%–80% protein, the highest among leguminous plants (Shewry et al. 1995). The different soybean products include soy milk, natto, tofu, soy sauce, soy flour, etc. Crude soybean protein has been widely used in food products such as infant and older adult nutritional formulas, meats, cereals, vegan products, etc., because of its high‐quality non‐dairy protein. However, it is not devoid of some health challenges, ranging from hay fever, asthma, and food allergies (Meinlschmidt et al. 2016). Soybean protein is known to be among the world's top 10 food allergies (Food Allergy Research Education 2023). For instance, it was reported to cause an allergic reaction in infants fed with formulas made with soy protein because of its high molecular weight (Sicherer and Sampson 2005). Food products whose functional properties can be enhanced for improved animal and human nutrition, health benefits, and with no deleterious effect, have therefore been the focus of emerging functional foods. Hydrolysis is one major way of breaking high molecular weight proteins into smaller ones (peptides or hydrolysates), which can lessen allergies and improve functional properties (Moure et al. 2005). Emulsification, solubility, foaming, and water–oil holding capacity are among the associated functional properties of protein peptides that enhance their bioactivities (Ajibola et al. 2016). Hydrolysis of food proteins by enzymatic methods, among other methods, is gaining attention in food processing and industry for enhancing food quality. One breakthrough recorded in this area is the replacement of egg white with whipping protein made from pepsin‐treated soy protein (Hrčková et al. 2002). The enzymatic method of protein hydrolysis, in contrast to heat and chemical methods, helps to prevent amino acid degradation, including cysteine, serine, and tryptophan, and the loss of food protein bioactivities (Hsu 2010). This process also improves the functional properties of food products and affords better advantages in the management of a wide range of human diseases (Arise et al. 2021; Okagu et al. 2021). In addition, the method has accounted for a high yield of protein (Ezequiel et al. 2016; Xiang et al. 2008). Protein hydrolysates are products obtained by hydrolyzing proteins (breaking down proteins into free amino acids) through various biological or chemical processes. While the chemical process of protein hydrolysis involves the use of acids (often called acid hydrolysis), that is, HCl, and bases (often called alkaline hydrolysis), for example, hydroxides of calcium, potassium, and sodium, to break down proteins into hydrolysates or peptides of lower molecular weights, the biological process of protein hydrolysis is mostly an enzymatic process. It involves the use of enzymes, for example, pepsin from animals, papain from plants, and alcalase from microorganisms, to break the peptide bonds of proteins into smaller peptides or hydrolysates. The biological process of protein hydrolysis is a natural process in the living system, for example, during digestion, and can also be applied industrially. Furthermore, food protein peptides or hydrolysates are preferred over synthetic products due to their endogenous internalization with cellular biomolecules and without any deleterious side effects (Festa et al. 2020; Lammi et al. 2019). The choice of enzyme for protein hydrolysis is significant in determining whether the hydrolysates exhibit functional properties, as this directly influences the characteristics of the end products (Zhang et al. 2020). Several studies have mostly employed the use of pepsin for protein hydrolysis (Badr et al. 2024; Olasehinde et al. 2023; Zhang et al. 2020; Ashaolu and Yupanqui 2017). The preference of pepsin to cleave aromatic and hydrophobic amino acids within proteins optimally at acidic pH to give a better yield of hydrolysate and protein recovery, its low bitter hydrolysate taste, and its cost‐effectiveness infer its choice among other enzymes for protein hydrolysis (Olasehinde et al. 2023; Ashaolu and Yupanqui 2017; Betts and Russell 2003). Nevertheless, the use of other proteolytic enzymes like alcalase, trypsin, and flavorzyme to develop hydrolysates has also been explored (Senadheera et al. 2023; Yan et al. 2016). While other proteases, such as subtilisin, chymotrypsin, papain, or bacillolysin, may share overlapping specificity, none retain robust hydrolytic activity at low pH, making pepsin uniquely suited to this process. Therefore, the selection of pepsin as the hydrolyzing enzyme in this study is based on its optimal activity in strongly acidic conditions (pH 1.5–3.5), which aligns with the simulated gastric environment and supports the release of biologically active peptides under conditions reflective of human digestion (Rawlings and Salvesen 2022). Some common sources of protein for hydrolysates are beans, mung beans, fish, wheat, shrimp, and soybeans (Ashaolu 2020). Soy protein hydrolysates (SPHs) are products of soybean protein hydrolysis. When crude soybean protein is hydrolyzed, the functional properties of the soy protein may be accelerated. SPHs obtained by enzymatic hydrolysis have been reported to regulate blood sugar levels in diabetic states and reduce weight gain in obesity (Gibbs et al. 2004), regulate blood pressure (Chiang et al. 2006), and also demonstrate anti‐hyperlipidemic and hypercholesterolemic activities (Hori et al. 2001; Yoshikawa and Takahashi 1993). Other studies have reported their immunomodulatory effects by boosting the immunoglobulin levels in rats, stimulating the proliferation of murine splenocytes, antimicrobial activity, antioxidant activity, and anticancer activity (Li‐Chan 2015; Ashaolu et al. 2017; Kiewit et al. 2018; Badr et al. 2024). Aside from the therapeutic value, SPHs have also been used as adsorbents, foaming, gelation, and emulsification agents (Hou and Zhao 2011; Martınez et al. 2009). In spite of several studies that purported the biological efficacies and physiological properties of SPHs, there is a paucity of information in the literature, particularly on the proximate composition of soy protein hydrolysate obtained by pepsin hydrolysis, as well as its cell viability and anti‐inflammatory activities. Therefore, this study aimed at investigating the proximate and amino acid composition of soy protein hydrolysate obtained by pepsin from soy protein isolate and its antioxidant, cell viability, and inhibitory effects on nitric oxide‐mediated inflammation in the raw cell line (Figure 1) for its application in drug and food products.

## 2 Materials and Methods

### 2.1 Materials

The soy protein isolate (a product of defatted soy flour) was commercially purchased from Wachsen Industry Company Limited, China. Pepsin, 1,1‐diphenyl‐2‐picrylhydrazyl (DPPH), 2,4,6‐tris (2‐pyridyl‐s‐triazine) (TPTZ), 2,2‐azobis (2‐amidino‐propane) dihydrochloride (AAPH), and fluorescein used in this study were products of Sigma Co. (St. Louis, MO, USA). Other chemicals and reagents used for this study were analytical grade and were also purchased commercially.

### 2.2 Methods

#### 2.2.1 Processing of Soybean Isolates and Enzymatic Hydrolysis

**FIGURE 2:**
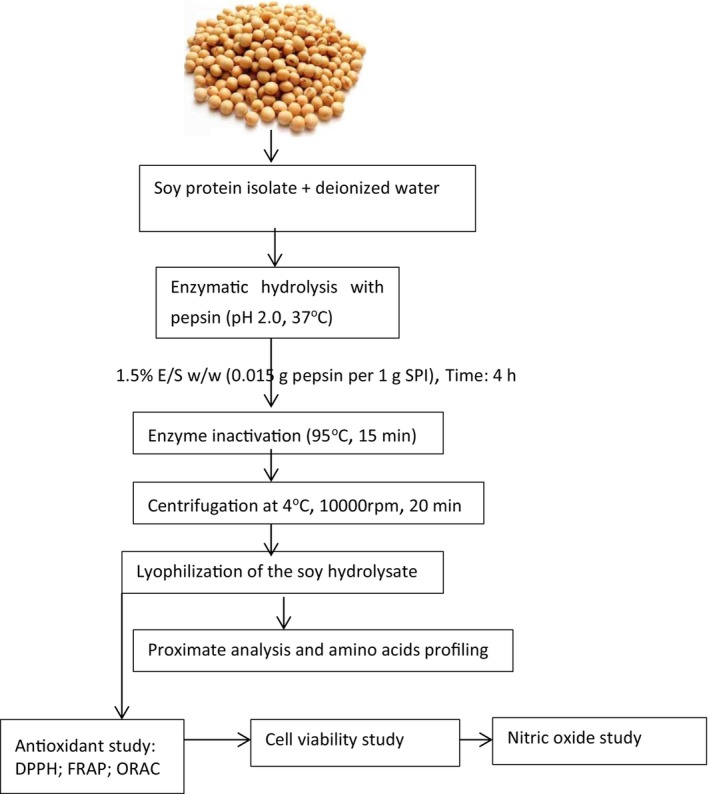
Flowchart illustrating the processing of soy protein isolate into hydrolysate by pepsin hydrolysis and the bioactivity assay. DPPH, 1,1‐diphenyl‐2‐picrylhydrazyl; FRAP, ferric reducing antioxidant power; ORAC, oxygen radical absorbance capacity; SPI, soy protein isolate.

The soy protein isolate (90.2% protein content) was processed for hydrolysis using an earlier method of Ashaolu and Yupanqui (2017) with slight modifications. SPI (10 g) was weighed and dissolved in 100 mL deionized water (1:10 w/v concentration) in a beaker. The solution was placed on a magnetic stirrer and stirred for 20 min at room temperature to form a homogenous suspension. The pH of the solution was adjusted to 2 by adding 1 M HCl, and incubated in a water bath for 15 min at 37°C to allow the protein to relax and partially unfold before enzyme hydrolysis. Enzyme hydrolysis was carried out using pepsin at an optimum temperature of 37°C and pH of 2 with HCl for 4 h using 1.5% E/S (w/w), as illustrated in Figure 2 below.

#### 2.2.2 Determination of Protein Recovery

At the end of the hydrolysis, the obtained SPH was centrifuged at 5000 rpm for 20 min at 4°C. The clear supernatant was collected for protein estimation by mixing 100 μL of the supernatant with 5 mL of Barford reagent (100 mg/L Coomassie Brilliant Blue G‐250, 85% (v/v) phosphoric acid, 95% ethanol). The mixture was then incubated at 25°C for 10 min, and absorbance was measured at 595 nm. A standard protein curve was prepared using BSA (1 mg/L) to estimate the protein concentration in mg/mL. A blank (consisting of Barford reagent and distilled water) and a control (consisting of distilled water + enzyme only) were prepared. The protein recovery, which measures the amount of soluble protein in the hydrolysate supernatant after hydrolysis compared to the total protein initially present in the SPI solution, was calculated using the formula of Xiang et al. (2008):

$$\text{Protein recovery}\left(\%\right)=\frac{\text{Protein in hydrolysate supernatant}}{\text{Initial protein content in}\;\mathrm{SPI}\;\text{solution}}\times 100$$

#### 2.2.3 SDS‐Page Electrophoresis

Soy protein isolate (SPI) and soy protein hydrolysate (SPH) were subjected to SDS‐PAGE (sodium dodecyl sulfate–polyacrylamide gel electrophoresis). The procedure of Laemmli (1970), which used a 12% acrylamide resolving gel and an acrylamide stacking gel (3%), was adapted. The protein samples were dissolved in electrophoresis sample buffer (4% SDS, 20% glycerol, 10% β‐mercaptoethanol, 0.125 M Tris–HCl, pH 6.8), and later 20 μg of the aliquots were loaded into an ELP 300 laboratory gel electrophoresis (Wincom Company, China). After electrophoresis, protein bands were stained with Coomassie brilliant blue R‐250 (Bio‐Rad, Richmond, CA, USA). The molecular weights of the protein bands were determined by comparing their mobility with molecular weight markers ranging from 5 to 270 kDa.

#### 2.2.4 Proximate Analysis and Profiling of Amino Acids From Soybean Protein Hydrolysate

The exact analysis of the SPH for protein, fat, total carbohydrate, ash, sugar, dietary fiber, and moisture contents was carried out using the standard procedure of Laemmli (1970). The Kjeldahl method was used to evaluate total nitrogen, which was subsequently used to determine crude protein content using a factor of 6.25 to multiply total nitrogen. The mineral (sodium, calcium, and iron) and vitamin constituents were determined using an atomic absorption spectrophotometer (AAS). For the profiling of amino acids, 1 mg of the SPH sample was hydrolyzed with 6 M HCl in a vacuum at a high temperature (110°C) for 20 h. This was followed by evaporating the hydrolysate to dryness. The dried SPH was dissolved in sodium tetraborate buffer (200 μL) and spun in a centrifuge. 100 μL of the supernatant was injected into an amino acid analyzer to profile the amino acids. These analyses were carried out at the Central Laboratory Company Limited in Songkhla, Hat Yai, Thailand. Energy (kcal) was estimated from Equation (1) below:

(1) $$\text{Energy}\;\left(\text{Kcal}\right)=\left(4\times \%\mathrm{CHO}\right)+\left(4\times \%\mathrm{CP}\right)+\left(9\times \%\mathrm{CL}\right)$$

where %CHO = % of carbohydrate; %CP = % of crude protein; and %CL = % of crude lipid.

#### 2.2.5 In Vitro Antioxidant Activity Assessment

##### 2.2.5.1 1,1‐Diphenyl‐2‐Picrylhydrazyl (DPPH) Assay

The radical scavenging activity of SPH for DPPH (1,1‐diphenyl‐2‐picrylhydrazyl) was determined by employing the procedure of Zheleva‐Dimitrova (2013) with a slight modification. Briefly, 200 μM of DPPH was prepared by dissolving 0.0197 g of DPPH in 250 mL of methanol. 100 mg/mL of the SPH was reconstituted with deionized water. The solution was centrifuged at 25°C and 3800 *g* for 10 min. The supernatant was collected as a stock solution and diluted serially (dilution factor of 10) with deionized water into concentrations of 10, 20, 30, 40, 50, and 60 mg/mL. A stock solution of ascorbic acid (60 mg/mL) used as the standard antioxidant compound was also prepared and made into different concentrations (10–60 mg/L). 50 μL of the SPH sample and ascorbic acid at each concentration were pipetted into a white transparent 96‐well plate, and 150 μL of the 200 μM DPPH was added to make a total volume of 200 μL. The mixture was vortexed for 30 s, incubated for 30 min, and absorbance was read at 517 nm in a microplate reader (BioTek PowerWave X; Gen5 1.09 software). This process was repeated thrice and was equally adopted for ascorbic acid. A control that consisted of deionized water and DPPH but with no sample was also prepared. The percentage inhibition of DPPH by SPH and ascorbic acid was calculated using Equation (2) below:

(2) $$\%\text{Inhibition}=\frac{\text{Absorbance of control}-\text{Absorbance of sample}}{\text{Absorbance of control}}\times 100$$

##### 2.2.5.2 Ferric Reducing Antioxidant Power Assay

The determination of ferric reducing antioxidant power for SPH was done by employing the procedure of Benzie and Strain (1996) with minor modifications. Stock solutions of SPH (100 mg/mL) and Trolox (60 mg/mL) were prepared and diluted accordingly with deionized water, as mentioned earlier, into different concentrations (1–6 mg/mL). The FRAP working reagent was freshly made by preparing and mixing acetate buffer (pH 3.6, 300 mM), 20 mM FeCl_3_.6H_2_O (iron III chloride), and TPTZ (10 mM) at a ratio of 10:1:1 before use. Fifty microliters each of the varying concentrations of the SPH sample and Trolox were pipetted into a white transparent 96‐well plate, and 150 μL of FRAP working reagent was added to give a total volume of 200 μL. The mixture was incubated at 37°C in the dark, and absorbance was measured in a microplate machine at 593 nm. This procedure was repeated in triplicate.

##### 2.2.5.3 Oxygen Radical Absorbance Capacity Assay

The oxygen radical absorbance capacity assay for SPH was conducted using the procedure of Ou et al. (2001) with slight modifications. Stock solutions of SPH (100 mg/mL) and ascorbic acid (60 mg/mL) were prepared as earlier mentioned, except that SPH was prepared with deionized water and later diluted with phosphate buffer 7.5 mM, pH 7.4, to make different concentrations (10, 20, 30, 40, 50, 60 mg/L). 50 μL of SPH and ascorbic acid, respectively, of the different concentrations, were pipetted into a black, non‐transparent 96‐well plate, and a fluorescein working solution 8.16 × 10^−5^ mM (150 μL) was added to each well. The mixture was first incubated in the dark in a microplate incubator without shaking at 37°C for 30 min. The fluorescence intensity was then measured at 45 nm (excitation) and 530 nm (emission) and recorded as F0 using a microplate reader (Thermo Scientific Varioskan LUX; S/N 3020–81,501, Skanlt RE 6.1.1 software). Then 50 μL AAPH (153 μM) was added to the mixture, and it was incubated again by shaking at 37°C. The intensity of the fluorescence was read again at 485 nm for excitation and 530 nm for emission every 2 min up to 1 h 30 min (46 cycles) and recorded as F2‐F92. A calibration curve for Trolox was prepared. The analysis was performed in triplicate. The oxygen radical absorbance capacity was expressed as μmol Trolox equivalent/100 g sample from the area under the curve using the Trolox standard.

#### 2.2.6 Cell Viability Assay

The activity of SPH on cell survival was evaluated by a colorimetric assay using 3‐(3,5‐dimethylthiazol‐2‐yl)‐2,5‐diphenyltetrazolium bromide (MTT) as described by Sae‐Wong et al. (2011), but with a slight modification. Murine macrophage (RAW264.7) was cultured in RPMI‐1640 (consisting of 10% fetal bovine serum (FBS), 100 U/mL of penicillin, and 100 μg/mL of streptomycin) with 5% carbon dioxide in a CO_2_ incubator at 37°C. After the cell growth reached 80% of the tissue culture ware, 0.25% trypsin‐ethylenediaminetetraacetic acid (EDTA) solution was used to wash cells from the tissue culture ware and counted to 5 × 10^5^ cells/mL. This was followed by pipetting 100 μL of the cell solution into each well of the 96‐well plate and incubating for 2 h. RPMI‐1640 (serving as standard) was replaced with 100 μL of the SPH solution at the different concentrations (0.5, 1.0, 2.5, 5, 10, 25 mg/mL), which was added to each well (RPMI‐1640 100 μL used as standard) and incubated for 24 h. Thereafter, 10 μL of MTT solution at a concentration of 5 mg/mL was added to a 96‐well plate and incubated at 37°C for 2 h. All solution was replaced with 200 μL of dimethyl sulfoxide (DMSO) to dissolve the formazan crystal, and absorbance was read at 570 nm. Cell viability was calculated in percentage using Equation (3) below. The concentration with cell viability higher than 80% was selected for the anti‐inflammatory properties:

(3) $$\%\text{of cell viability}=\frac{\text{Absorbance of control}}{\text{Absorbance of standard}}\times 100$$

#### 2.2.7 Anti‐Inflammatory Activity of SPH in Nitric Oxide Oxidative Stress‐Mediated Inflammation

The anti‐inflammatory activity of SPH was evaluated by the method described by Sae‐Wong et al. (2011) with a slight modification. RAW‐264.7 (murine macrophage) cells were cultured in RPMI‐1640 (Roswell Park Memorial Institute) containing fetal bovine serum (10%), penicillin (100 U/mL), and streptomycin (100 μg/mL) with 5% carbon dioxide (CO_2_ incubator) at 37°C. 100 μL of cell solution was placed in a 96‐well plate and incubated for 2 h. Thereafter, RPMI‐1640 was substituted with a solution of lipopolysaccharide (200 μg/mL) and 100 μL of RPMI‐1640 per well, used as a standard. This was followed by the addition of 100 μL each of different concentrations (10, 5, 2.5, and 1 mg/mL) of SPH to the plate, except for the control and blank, which used 100 μL of RPMI and were incubated at 37°C for 24 h. After incubation, 100 μL of each well's content was transferred to another well for anti‐inflammatory evaluation, which employed 100 μL of Griess reagent per well. This was followed by absorbance measurements at 570 nm. Nitrite concentration was considered as the percentage nitric oxide production inhibition as calculated in Equation (4) below:

(4) $$\mathrm{NO}\;\text{inhibition}\left(\%\right)=\frac{\left({\mathrm{Abs}}_{C}-{\mathrm{Abs}}_{\mathrm{Bc}}\right)-\left({\mathrm{Abs}}_{S}-{\mathrm{Abs}}_{\mathrm{Bs}}\right)}{\left({\mathrm{Abs}}_{C}-{\mathrm{Abs}}_{\mathrm{Bc}}\right)}\times 100$$

where Abs_C_ = absorbance of control (RPMI + LPS); Abs_Bc_ = absorbance of blank (RPMI); Abs_S_ = absorbance of sample solution (Sample + LPS); Abs_Bs_ = absorbance sample blank (Sample + RPMI).

### 2.3 Statistical Analysis

The experiment was performed in triplicate for accuracy. The data obtained were expressed as the mean ± standard deviation (SD) and were analyzed using GraphPad Prism software version 6.0. A significant difference at *p* < 0.05 was determined by analysis of variance (ANOVA) using Tukey's test.

## 3 Results

### 3.1 Proximate Analysis and Amino Acids Composition

The nutritional composition and protein recovery of the SPH are presented in Table 1. The protein recovery was 89.71%. The SPH contained carbohydrate (13.66%), protein content (71.18%), total fat (1.92%), dietary fiber (13.44%), moisture (2.45%), and ash content (10.79%) per 100 g, and a total energy supply of 356.64 kcal. The SPH was also found to contain sodium (5496.63 mg), calcium (85.528 mg), iron (6.535 mg), and vitamin B1 (1.10 mg). Table 2 depicts the amino acid profile of the SPH obtained by pepsin hydrolysis in comparison with the crude soy protein isolate. The SPH contained 45.36% and 54.70% essential and non‐essential amino acids, respectively, with glutamate (18.35%) constituting the major amino acid and cysteine (0.13%) the least abundant amino acid. Hydrophilic amino acids dominated the amino acids, with a total of 60.13% compared to hydrophobic amino acids with a total of 38%. There were varying amounts of amino acid composition in the crude SPI and SPH. Only cysteine, glutamine, and methionine appeared in higher amounts in the SPI (0.18%, 2.89%, and 2.43%, respectively) than in the SPH (0.13%, 2.48%, and 1.53%), while asparagine was not detected in either the SPI or SPH (Table 2).

**TABLE 1:** Nutritional composition of soy protein hydrolysate obtained by enzymatic hydrolysis.

Test item	% Composition per 100 g sample
Protein recovery	89.7%
Energy	356.64 (kcal)
Energy from fat	17.28
Total fat	1.92
Saturated fat	1.00
Cholesterol	Not detected
Protein content	71.18
Total carbohydrate	13.66
Dietary fiber	13.44
Sugar	0.00
Sodium (mg)	5496.63
Vitamin A (μg)	Not detected
Vitamin B1 (mg)	1.10
Vitamin B2 (mg)	Not detected
Calcium (mg)	85.528
Iron (mg)	6.535
Ash content	10.79
Moisture content	2.45

Abbreviations: μg, microgram; g, gram; kcal, kilocalories; mg, milligram.

**TABLE 2:** Amino acid profile of soy protein isolate (SPI) and soy protein hydrolysate SPH.

Amino acid	% composition in SPI	% composition in SPH
Alanine	3.02	4.78
Arginine^a^	4.08	9.27
Aspartate	4.51	10.13
Cystine	0.59	1.87
Glutamate	3.64	18.35
Glycine	2.38	3.57
Histidine^a^	1.42	3.63
Isoleucine^a^	4.15	3.84
Leucine^a^	4.24	6.78
Lysine^a^	3.90	5.69
Methionine^a^	2.43	1.53
Phenylalanine^a^	3.24	5.68
Proline	3.72	6.62
Serine	3.25	3.67
Threonine^a^	2.95	3.74
Tryptophan^a^	0.74	1.19
Tyrosine	2.31	3.10
Valine^a^	2.30	4.01
Asparagine	Not detected	Not detected
Cysteine	0.18	0.13
Glutamine	2.89	2.48
TOTAL	100	100
TEAA (SPH)		45.36
TNEAA (SPH)		54.7
THAA (SPH)		38
!THAA (SPH)		60.13

^a^ EAA = essential amino acids; TEAA = total essential amino acids; THAA = total hydrophobic amino acids; !THAA = total hydrophilic amino acids TNEAA = total non‐essential amino acids.

### 3.2 Molecular Weight of Soy Protein Isolate and Hydrolysate

**FIGURE 3:**
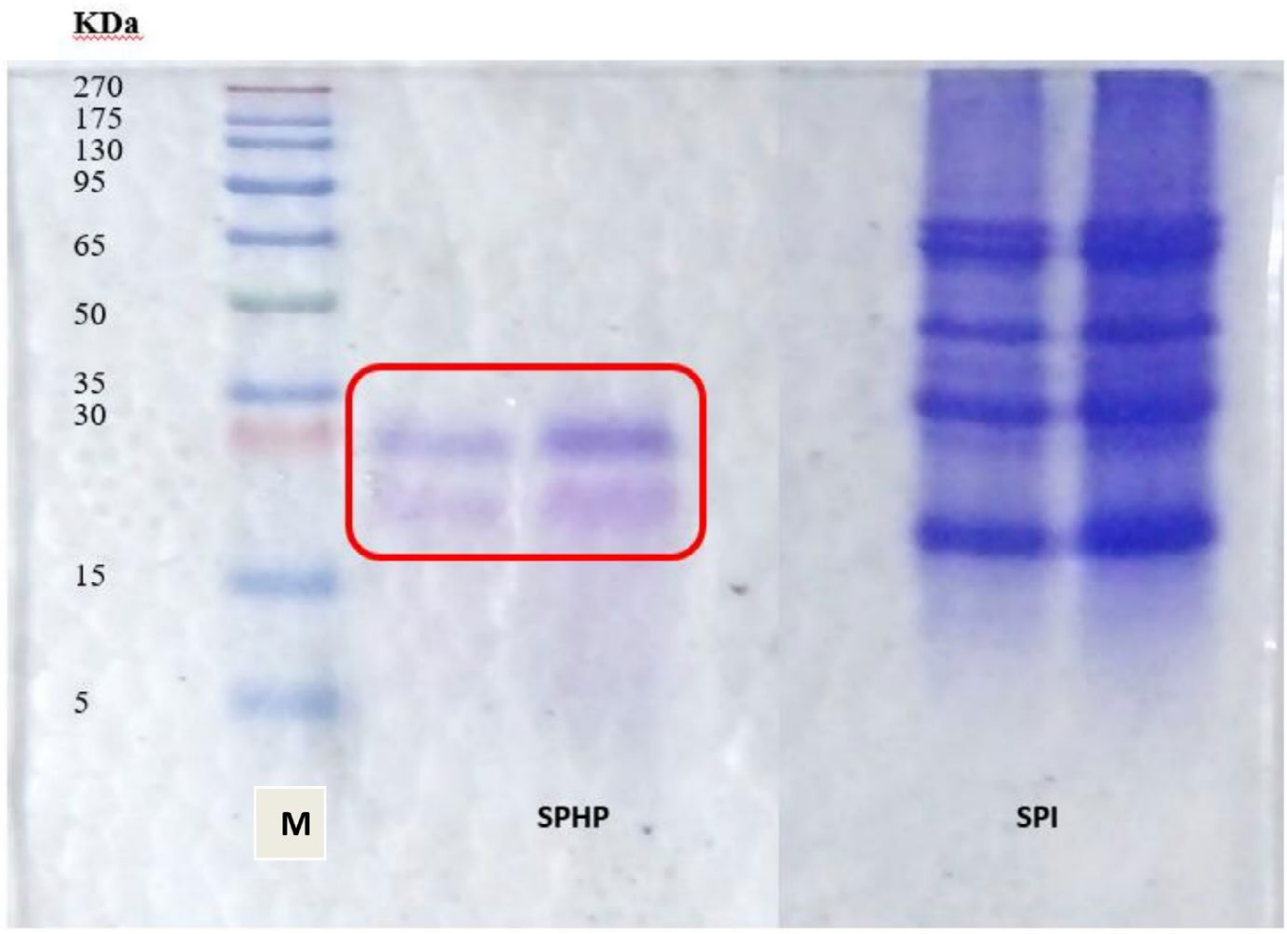
Sodium dodecyl sulfate polyacrylamide gel electrophoresis (SDS‐PAGE) of SPHP and SPI. KDa, kilo Dalton; M, marker protein; SPHP, soy protein hydrolyzed with pepsin; SPI, soy protein isolate.

Figure 3 shows the molecular weight of the SPI and SPH obtained by sodium dodecyl sulfate–polyacrylamide gel electrophoresis (SDS‐PAGE). The SDS‐PAGE lanes showed the molecular weight of the hydrolysate to be < 20 kDa and SPI to be > 65 kDa, as indicated by the intensity of the bands' color.

### 3.3 Antioxidant Activity of Soy Protein Hydrolysate

**FIGURE 4:**
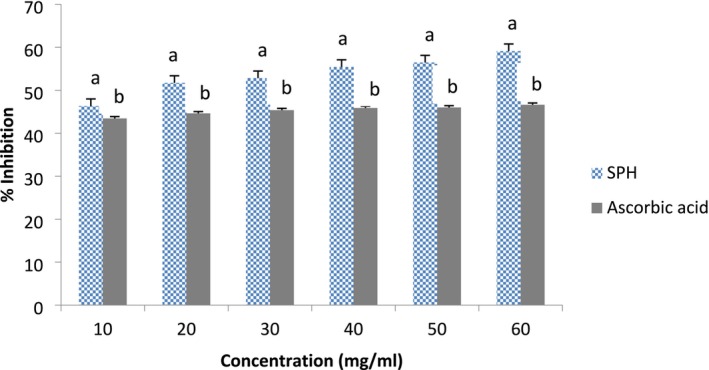
DPPH (1,1‐diphenyl‐2‐picrylhydrazyl) scavenging activity of soy protein hydrolysate and ascorbic acid. Data are mean ± SD in triplicate. Bars with different alphabets are significantly different (*p* < 0.05).

**FIGURE 5:**
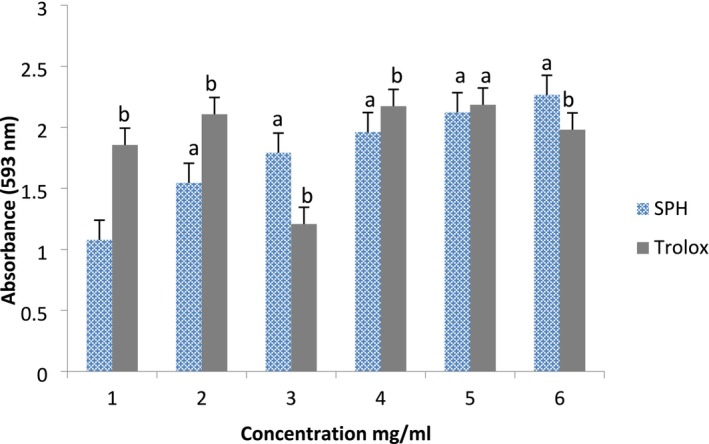
Ferric reducing antioxidant power of soy protein hydrolysate and Trolox. Data are mean ± SD in triplicate. Bars with different alphabets are significantly different (*p* < 0.05).

The DPPH radical scavenging activity and ferric reducing antioxidant power of the SPHs are presented in Figures 4 and 5, respectively. SPH demonstrated a concentration‐dependent scavenging activity for DPPH; that is, the highest inhibitory activity (61.42%) for DPPH was recorded at the highest concentration (60 mg/mL), and the least inhibitory activity (47.56%) was recorded at the lowest concentration (10 mg/mL) compared to ascorbic acid, the reference antioxidant compound, which inhibited DPPH in a similar proportion at the different concentrations investigated (Figure 4). Similar to the DPPH result, SPH also demonstrated concentration‐dependent ferric reducing antioxidant power (FRAP) when compared to the reference antioxidant compound (Trolox). The highest FRAP for the SPH was recorded at the highest concentration (6 mg/mL) investigated. It was noticed that at 539 nm, the SPH only demonstrated significantly higher (*p* < 0.05) FRAP (2.27 units) than the reference antioxidant compound (1.98 units). However, at lesser concentrations, Trolox demonstrated significantly higher (*p* < 0.05) FRAP than the SPH (Figure 5). In Table 3, the SPH oxygen radical absorbance capacity expressed in Trolox equivalent was 3647.2 ± 14.2 μmol TE/g compared to ascorbic acid with 2567.3 ± 7.2 μmol TE/g.

**TABLE 3:** Oxygen radical absorbance capacity of soy protein hydrolysate.

Sample	Concentration
SPH	3647.2 ± 14.2 μmol TE/g
Trolox	2567.3 ± 7.2 μmol TE/g

*Note:* Data are mean ± SD in triplicate.

Abbreviations: SPH, soy protein hydrolysate; TE, Trolox equivalent.

### 3.4 Cell Viability and Anti‐Inflammatory Effect of Soy Protein Hydrolysate

**FIGURE 6:**
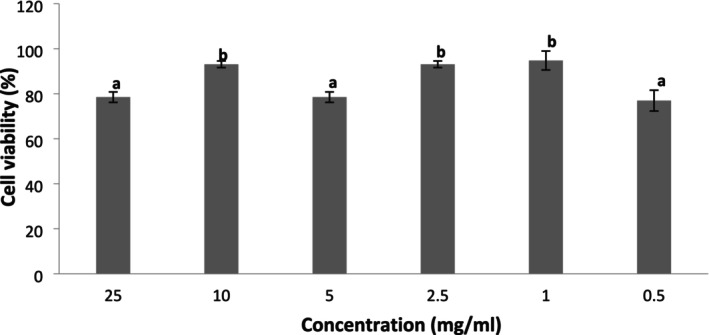
Cell viability effect of soy protein hydrolysate on RAW‐264.7 cells after 4 h incubation. Data are mean ± SD in triplicate. Bars with different alphabets are significantly different (*p* < 0.05).

**FIGURE 7:**
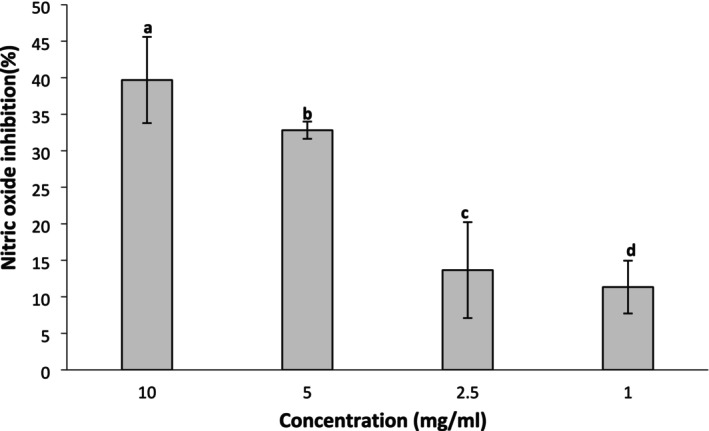
Effect of soy protein hydrolysate on nitric oxide inhibition in RAW‐264.7 cell. Data are mean ± SD in triplicate. Bars with different alphabets are significantly different (*p* < 0.05).

The cell viability effect of SPH was investigated at different concentrations using the MTT colorimetry assay, as presented in Figure 6. SHP promoted over 80% cell survival or viability at 10, 2.5, and 1 mg/L and was not significantly different (*p* > 0.05) from each other. At concentrations of 25, 5, and 0.5 mg/L, the cell survival rate was approximately 80% after treatment with SPH, and was not significantly different (*p* > 0.05) from each other. Figure 7 depicts the inhibitory effect of SPH on nitric oxide (NO) production. SPH demonstrated a concentration‐dependent nitric oxide inhibition, with the strongest inhibitory effect (40%) recorded at the highest concentration (10 mg/mL) and the least inhibitory effect (10%) recorded at the lowest concentration (1 mg/mL).

## 4 Discussion

The protein recovery (89.7%) of the SPH from the pepsin‐treated SPI at 1.5% E/S w/w for 4 h, pH 2.0, and a temperature of 37°C in this study was comparable with the protein recovery of 89.3% reported by Ashaolu and Yupanqui (2017) under similar conditions of hydrolysis, but higher than the 87.7% reported by the same authors using alcalase. Protein recovery describes the proportion of protein retained after hydrolysis relative to the amount of protein originally present in the crude sample. The high protein recovery by pepsin‐treated SPH at 1.5% E/S makes prospective commercial applications cost‐effective. Similarly, protein hydrolysates generated using pepsin as the hydrolyzing agent in other studies also yielded high protein hydrolysates (Olasehinde et al. 2023; Dia et al. 2014; Zhong et al. 2007). Although many factors influence protein recovery during hydrolysis, the type and concentration of the enzyme contribute significantly (Ashaolu and Yupanqui 2017). The SDS‐gel electrophoretic analysis from this study revealed that pepsin hydrolysis of SPI produced a hydrolysate of smaller molecular weight (< 20 kDa), when compared to the crude SPI of higher molecular weight (> 65 kDa). The small molecular weight of the SPH in this study is in consonance with Badr et al. (2024). It has been established that enzymatic hydrolysis of proteins results in peptides or hydrolysates of smaller molecular weight with less secondary structure. This property further promotes SPH solubility and intestinal absorption compared to intact proteins (Ziegler et al. 1998). The proximate analysis of the SPH also revealed high protein content (71.18%). The protein content was higher than the protein content reported for different non‐hydrolyzed soy protein products (soy protein concentrate, full‐fat soy protein, defatted soy flour, and raw soy flour) by other studies (Badr et al. 2024; Robbani et al. 2022). Additionally, the protein and ash contents for this study were higher than 50.61% and 4.78%, respectively, reported by Islam et al. (2022), while the moisture and total fat contents were lower than 7.66% and 10.07%, respectively, from the findings of Islam et al. (2022) for SPH obtained using alcalase. This variation could be attributed to the sample preparation method, the type or nature of the enzyme employed for hydrolysis, and the degree of hydrolysis employed. Protein and dietary fiber contribute significantly to the management of diabetes mellitus.

Proteins are necessary for body growth and cell repair. Moreover, plant sources of proteins, such as SPH, can effectively bind to body cell receptors to promote glucose (blood sugar) utilization, unlike animal protein sources. The protein content of SPH in this study not only suggests SPH as a rich source of protein but could also promote the utilization of glucose in diabetic conditions as a plant‐based protein. More importantly, other beneficial health effects include impeding chronic inflammatory diseases, gut epithelial protection, and lowering the concentrations of low‐density lipoproteins (Sadeghi et al. 2020; Bitzer et al. 2017). Dietary fiber, as one of the nutrients estimated in this study, is known to prevent fat storage, slow down carbohydrate digestion, and promote insulin. This implies that SPH could be relatively employed in blood sugar control, weight management, and reducing the incidence of coronary diseases since the dietary fiber content of the SPH is over 13%, which is more than three times the Thai recommended dietary intake. The high ash content noticed in this study also suggests SPH as a good source of minerals. Further investigation on the mineral constituents of the SPH revealed considerable amounts of minerals such as sodium, calcium, and iron to be present. The calcium value was higher, while the sodium value was lower than those reported by Robbani et al. (2022) for different crude soy proteins. Macro‐minerals are essential nutrients for the management of mineral deficiency‐related diseases such as osteoporosis, anemia, and coronary diseases (Saliu et al. 2023). Sodium can promote muscle and nerve cell development and also regulate blood pressure and volume (Saliu et al. 2023). Calcium is necessary for blood clotting, muscle contraction, tooth development, and bone mineralization (Saliu et al. 2023). The iron content for this study was lower than the iron content reported by Robbani et al. (2022) for soy protein concentrate (10.2 mg), full‐fat soy protein (19.01 mg), and defatted soy flour (10 mg per 100 g) but higher than the 6.23 mg/100 g reported for raw soy flour by the same authors. Iron, like calcium, can promote tooth and bone development. Iron is also strongly recognized in the treatment of anemic conditions. In addition, vitamins and minerals act as cofactors for many enzymes involved in metabolic processes. Therefore, the presence of these minerals and vitamins in SPH is beneficial to human health. Carbohydrates, proteins, fats, vitamins, and minerals are organic molecules that constitute a balanced diet; thus, the presence of these nutrients in the SPH suggests the SPH as a source of a balanced diet. Additionally, the considerable fat content of the SPH suggests that SPH can enhance food flavors when employed in the food industry.

This study reported an appreciable protein content and an increase in amino acid composition of the SPH. This may be attributed to the hydrolysis improving solubility by the enzymatic ability of pepsin to cleave more of the aromatic amino acid residues of protein to liberate soluble peptides and free amino acids into the supernatant fraction of the hydrolyzed SPI. This mechanistic process further enhances protein detectability in colorimetric assays employed for protein content determination, resulting in higher measurable protein concentration post‐hydrolysis (Wu et al. 2023). A total of 19 out of 20 known standard amino acids were primarily present in the SPH, with glutamate and aspartate being the most abundant and cysteine being the least abundant, while asparagine was absent in both the SPH and SPI. The previous study by Ashaolu and Yupanqui (2017) also reported a total of 19 amino acid constituents for unfractionated soy hydrolysate made from the pepsin hydrolysis model. Glutamate and aspartate are important amino acids that enhance food palatability (Islam et al. 2022). The absence of asparagine in the SPH is a reflection of its absence in the crude SPI, and it is consistent with the report of Ashaolu and Yupanqui (2017). The individual amino acid compositions in this study were higher than those reported by Islam et al. (2022). In a similar vein, the amino acid compositions of SPH from our study were higher than those documented by Robbani et al. (2022) for crude soy protein isolates. The total hydrophobic amino acid constituent for this study was 38%, while the total hydrophilic amino acid constituent was 60.13%. The higher percentage aggregate for hydrophilic amino acid residues is an indication of the highly soluble nature of the SPH, which will promote peptide absorption and digestibility as a functional ingredient for food products and formulations. According to Dash and Ghosh (2017), the good solubility of peptides is not only responsible for the smooth consistency of food products but also enhances their attractive appearance. Notably in our study is the higher percentage composition for 15 out of the 19 amino acids (Ala, Arg, Asp, Glu, Gly, His, Leu, Lys, Phe, Pro, Ser, Thr, Try, Tyr, and Val) in the SPH when compared to the SPI. Likewise, Ashaolu and Yupanqui (2017) also reported a higher percentage aggregate of amino acid compositions in SPH compared to SPI. This suggests that enzyme treatment using pepsin as a hydrolysis agent enhances the amino acid composition of peptides or hydrolysates. According to the submission of Anal et al. (2013), enzymes employed in protein hydrolysis improve the functional attributes of foods and the maintenance of their integrity. The enhanced nutrients and amino acids of SPH further suggest it SPH as an alternative to crude soy proteins. It also suggests it as a plant‐based replacement for animal‐based proteins since the digestibility‐corrected amino acid score has been reported to be the same for both plant‐based and animal‐based proteins (Singh et al. 2014). The SPH also demonstrated a higher percentage aggregate (54.70%) of non‐essential amino acids and a lower percentage aggregate (45.36%) of essential amino acids. This is a reflection of the percentage aggregate for both classes of amino acids in the SPI; however, each amino acid was appraised following pepsin hydrolysis. This is consistent with the report of Badr et al. (2024).

The essential amino acid composition for this study was above the FAO/WHO requirements for children and adults (FAO 2007). Essential amino acids are amino acids that are not synthesized in the human body but can be derived exogenously and are essentially required for growth and the proper functioning of the body. Therefore, their presence in higher composition in the SPH following enzymatic hydrolysis places it SPH as a promising emerging functional food substitute in the food industry. In contrast to our results, Ashaolu and Yupanqui (2017) reported higher values of Asp, Glu, His, Phe, Tyr, Try, and Val for soy protein hydrolysates SPH using pepsin. This disparity can be attributed to the physicochemical conditions and degree of hydrolysis adopted for the enzymatic hydrolysis of the soy protein, insoluble aggregates, and water loss due to lyophilizing (freeze‐drying) the hydrolysate. According to Vastag et al. (2010), enzyme type, enzyme concentration, hydrolysis time, and temperature are important hydrolytic conditions that affect the functional properties of peptides. Chemical reactions such as protein denaturation and Maillard reactions also cause changes in amino acid compositions in the hydrolytic state (Ashaolu and Yupanqui 2017). Cysteine was present in our study but absent in the report of Ashaolu and Yupanqui (2017). Cysteine, in conjunction with tryptophan and serine, plays significant roles in retaining the biological activities of food proteins; it is therefore suggested that their loss in food products be avoided (Hsu 2010). It is evident from this study that the SPH retained these three amino acids and also enhanced their compositions following pepsin hydrolysis. This further suggests that enzymatic hydrolysis is a promising technology for improving the functional features of food products. According to some reports, the enzymatic method of protein hydrolysis can ensure better nutritional and chemical values of food products, unlike chemical processes that utilize acids or bases, which are difficult to control and can also result in little or total reduction of some vital amino acids and even form some unusual amino acids, including lysinoalanine or lanthionine in peptides (Castro et al. 2011).

The concentration of cysteine as the least abundant amino acid in the SPH, which was also reflected in the SPI for this study, aligns with the fact that sulfur‐containing amino acids are considered to be limited in soybean products (Li et al. 2020). In an animal experimental study conducted by Ashaolu et al. (2017), SPH at different concentrations (5–15 mg), when administered to experimental animals for 2 weeks, caused drastic weight gain in experimental rats compared to their counterparts administered crude soy protein. This suggests that SPHs can promote growth and development in humans when replaced with intact protein, since the pepsin hydrolysis employed in this study enhanced the amino acid compositions of the SPH, which appeared in a lesser amount in the crude SPI. Oxidative stress is a major contributor to the pathogenesis of many diseases. Excessive generation of reactive oxygen species (ROS), which include singlet oxygen, superoxide ions, peroxides, hydrogen peroxide, etc., in the biological system induces oxidative stress when they outweigh endogenous antioxidants (antioxidant enzymes and glutathione) designed to combat and defend against radical effects. In addition, ROS are the major causes of lipid degradation and peroxidation of food and cosmetic matrices (Puozo et al. 2016). Exogenous antioxidants from plant sources are most often used to boost the endogenous antioxidant system in order to effectively ameliorate oxidative stress and damage induced by ROS by forming stable products that terminate the radical chains. Hence, the antioxidant activity of the SPH was investigated in this study. Different methods have been used to evaluate the antioxidant activity of a food product in vitro; however, DPPH and FRAP assays, which assess electron mitigations, and the ORAC assay, which assesses hydrogen atom transfer, are among the most common methods. The SPH demonstrated a concentration‐dependent scavenging activity for the DPPH radical. In the DPPH assay, the SPH expressed over 50% DPPH inhibition at higher concentrations (20–60 mg/mL) and was above ascorbic acid, the reference antioxidant compound. The DPPH method is the easiest method for investigating an antioxidant agent because the DPPH radical, which appears as a violet color in the assay, and in the presence of an antioxidant, receives a hydrogen atom to be converted to a non‐radical (DPPH + H), characterized by a yellow color (Saliu et al. 2020). Similar to the DPPH result, SPH also demonstrated concentration‐dependent ferric reducing antioxidant power when compared to the reference antioxidant compound (Figure 4). The highest FRAP absorbance for the SPH (2.27 units) was measured at the highest concentration (6 mg/mL) of the SPH and was significantly higher (*p* < 0.05) than the absorbance (1.98 units) for the reference antioxidant compound at the same concentration. In the FRAP assay, higher absorbance indicates higher reducing power. SPH also recorded a higher ORAC value than the reference antioxidant compound. From all indications, the SPH demonstrated antioxidant activity against DPPH and ferric radicals, thus suggesting the potential of the SPH to break radical chains and prevent cellular or oxidative damage that could result in pathological conditions. Though it is difficult to clarify the structure‐and‐activity relationship for peptides, particularly for antioxidant activity., we suggest that the antioxidant activity may be linked to the hydrogen‐donating nature of the hydroxyl groups in the hydrophobic amino acids of the SPH, acting in synergy with other reducing electrons of the SPH in executing the antioxidant activity. The radical scavenging activities of peptides are partly dependent on the nature of the peptides, among which are amino acid composition, molecular weight, size, and hydrophobicity (Girgih et al. 2015). However, some studies have also suggested other mechanisms for the antioxidant activity of protein peptides but have been correlated mostly to the hydroxyl group of aromatic amino acids (his, phe, try, and tyr), hydrophobic amino acid residues, and their positioning in the peptide sequence because of their ability to stabilize and scavenge free radicals more than their hydrophilic counterparts (Ambigaipalan and Shahidi 2017; Girgih et al. 2015; Cumby et al. 2008). Also, the strong reducing power of hydrolysates is associated with the availability of electrons or hydrogen atoms due to the release of peptides during protein hydrolysis (Chalamaiah et al. 2015). Besides the hydrophobicity, though the specific peptides and the amino acid sequences of the SPH were not identified in this study, the spatial structure of amino acid residues and their interactions in hydrolysate sequences have been suggested to play a significant role in the antioxidant property (Eftekharzadeh et al. 2010). Metal ion chelation, redox reactions, and inhibition of unsaturated fatty acid autoxidation are also part of the mechanisms by which peptides demonstrate antioxidant activity (Girgih et al. 2015; Senadheera et al. 2023). The radical scavenging activity demonstrated by the SPH in this study indicates that SPH could be applied as a natural antioxidant of exogenous source in the food industry to complement the endogenous antioxidant system in combating oxidative stress and its related diseases. Additionally, bioactive peptides (hydrolysates) from other protein sources, such as sea cucumber (Senadheera et al. 2023), thread fin bream surimi by‐products (Wiriyaphan et al. 2012), *Cyprinus carpio* (carp roe) (Chalamaiah et al. 2015), and cold fish (Girgih et al. 2015) have also been confirmed to show antioxidant activities against free radicals.

The cytotoxicity or cell viability assay is the method usually adopted to identify the potential of an agent to cause raw cell line destruction or promote cell growth. A high percentage of cell growth (viability), as high as 65%, by an agent connotes lower cytotoxicity and is an indicator of good cell health and vice versa (Suksanga et al. 2023; Matsuda et al. 2003). It is indicative from this study that SPH did not induce cell line destruction at all concentrations investigated, since up to 80% of the cells were recovered following the introduction of the SPH; thus, SPH may be considered safe. This corroborates the previous study of Ashaolu and Yupanqui (2017), who reported no cytotoxic effect in the raw cell line in vitro, with over 80% cell viability recorded after the introduction of SPH in the cell culture media. Further in vivo findings by Ashaolu et al. (2017) in an animal model also corroborate the safety of SPH, as rats fed a high dose of SPH up to 5000 mg/kg b.w. manifested no toxic symptoms and no significant changes in the skin, hair, and there was no mortality in the rat subjects. Similarly, the study of Korhonen (2009) also reported the safety of soybean peptides with no deleterious effects. Other experimental studies conducted on hydrolysates of other protein sources, such as casein, also affirmed the safety of protein peptides (Papineni et al. 2017; Anadón et al. 2010; Kobayashi et al. 2004). Although the SPH demonstrated a concentration‐dependent nitric oxide inhibition, however, this effect was mild, causing approximately a 40% inhibitory effect on NO. Nitric oxide plays a dual physiological role in the biological system, one as a free radical and the other as a vasodilator. High nitric oxide generation as a free radical is linked to inflammatory disorders and other physiopathological conditions (Spiller et al. 2019). Therefore, reducing or inhibiting the production of NO is considered a primitive measure for inflammation. Reduction of nitric oxide is interpreted as no inflammation or no cell death. Cell viability tests, which determine cell survival, are usually recommended to be evaluated before conducting anti‐inflammatory tests. Hence, the concentrations of 10, 5, 2.5, and 1 mg/mL were adopted in this study to evaluate the nitric oxide inhibitory activity of SPH following the outcome of the cell viability test, where over 80% cell survival or viability was recorded at these concentrations.

From our findings, SPH demonstrated anti‐inflammatory activity as it caused an appreciable inhibition of nitric oxide at the highest concentration investigated. Our study showed a higher anti‐inflammatory activity in inhibiting NO at the highest concentration than the report of Badr et al. (2024). This variation may be attributed to the nature and molecular weight of the hydrolysates generated, and the degree of hydrolysis employed. The mild anti‐inflammatory activity of the SPH may suggest an antagonistic effect of the hydrophobic and hydrophilic amino acids in the SPH or through other mechanisms. A high amount of hydrophobic amino acids contributes to the amelioration of oxidative stress‐mediated diseases, including inflammatory responses. The anti‐inflammatory activity demonstrated by the SPH may also not be disconnected from the antioxidant activity of the SPH, thus suggesting the potential of SPH in mediating inflammatory responses. Other studies have also related the anti‐inflammatory activity of some hydrolysates obtained by pepsin hydrolysis to their antioxidant activities. The study of Oluwajuyitan et al. (2025) revealed that pepsin hydrolysate from fava bean demonstrated antioxidant and anti‐inflammatory activities, as well as better arginase, angiotensin‐converting enzyme, acetylcholinesterase, and α‐amylase inhibitory activities than hydrolysates obtained from other proteases (trypsin, alcalase, chymotrypsin, and thermoase). In light of the results obtained in this current study, soybean protein hydrolysate could serve as an emerging functional food ingredient for food products to substitute intact or crude proteins due to the excellent nutritional quality and biological activities that support animal and human health.

## 5 Conclusion

This study demonstrated that enzymatic hydrolysis of crude SPI using pepsin significantly improved its nutritional composition, reduced its molecular weight, and enhanced its biological activity. The resulting SPH exhibited a high protein recovery yield, a favorable balance of essential and non‐essential amino acids, and considerable mineral and energy content, indicating its potential as a nutritionally rich alternative to intact SPI and effective commercial application. Furthermore, SPH showed notable antioxidant activity through various radical scavenging assays and effectively inhibited nitric oxide production in LPS‐stimulated RAW 264.7 macrophages while promoting high cell viability. These findings underscore the potential of SPH obtained by pepsin hydrolysis as a functional food ingredient with health‐promoting properties.

### 5.1 Specific Directions for Future Study

The study recommends the need to characterize the bioactive peptides using advanced techniques such as LC–MS/MS or HPLC to identify and sequence the bioactive peptides responsible for antioxidant and anti‐inflammatory effects. Animal model studies or clinical trials should be conducted to validate the safety, bioavailability, and functional efficacy of soy protein hydrolysate in living systems, while the in vitro studies have already provided promising results. It is also important to carry out dose optimization and stability to investigate the stability, optimal dosage, and shelf life of SPH under various food processing and storage conditions. Sensory evaluation, such as taste, odor, and overall palatability of the hydrolysate, which are important for consumer acceptance, should also be assessed.

## Author Contributions

**Oluwafemi Ayodeji Idowu:** formal analysis (lead), methodology (lead), project administration (equal), writing – original draft (lead), writing – review and editing (lead). **Chutha Takahashi Yupanqui:** conceptualization (lead), funding acquisition (lead), project administration (equal), supervision (lead).

## Conflicts of Interest

The authors declare no conflicts of interest.

## Data Availability

Data are available on request due to privacy/ethical restrictions.
